# BET proteins target murine leukemia virus integration to transcription start sites

**DOI:** 10.1186/1742-4690-10-S1-O19

**Published:** 2013-09-19

**Authors:** Amit Sharma, Ross Larue, Matthew Plumb, Nirav Malani, Frances Male, Alison Slaughter, Jacques Kessl, Nikoloz Shkriabai, Elizabeth Coward, Sriram Aiyer, Patrick Green, Li Wu, Monica Roth, Frederic Bushman, Mamuka Kvaratskhelia

**Affiliations:** 1 Center for Retrovirus Research and College of Pharmacy, The Ohio State University, Columbus, Ohio, USA; 2 Department of Microbiology, University of Pennsylvania Perelman School of Medicine, Philadelphia, Pennsylvania, USA; 3 Department of Pharmacology, University of Medicine & Dentistry of NJ-Robert Wood Johnson Medical School, Piscataway New Jersey, USA; 4 Center for Retrovirus Research and Department of Veterinary Biosciences, The Ohio State University, Columbus, Ohio, USA

## Background

The selection of chromosomal targets for retroviral integration varies markedly, tracking with the genus of the retrovirus, suggestive of specific targeting by cellular factors. Gamma-retroviral murine leukemia virus (MLV) DNA integration into the host genome is favored at transcription start sites, but the underlying mechanism for this preference is unknown. The molecular mechanisms of MLV integration are of particular significance due to the fact that MLV-based vectors are used for human gene-therapy. In clinical trials the use of MLV-based vectors to correct primary immunodeficiencies has been curative, but adverse events have occurred that are associated with the insertional activation of proto-oncogenes. Therefore the identification of cellular factors that control MLV integration may provide mechanistic clues to facilitate the development of safer gene-therapy vectors.

## Materials and methods

We used affinity capture coupled with mass spectrometry to identify cellular binding partners of MLV integrase. The direct interactions between MLV integrase and its cellular binding partners were characterized using purified recombinant proteins. Furthermore, we examined the effects of the purified cellular proteins in *in vitro* integration assays catalyzed by MLV integrase. To dissect the role of the cellular proteins in MLV integration site selection, we analyzed the distribution of 11,968 unique integration sites in cells treated with the selective inhibitor JQ-1 or a pool of siRNAs targeting the cellular proteins by 454 pyrosequencing.

## Results

We have identified BET proteins (Brd2, 3, 4) as principal cellular binding partners of MLV integrase [1]. We show that purified recombinant Brd4 binds with high affinity to MLV integrase and stimulates correct concerted integration *in vitro*. Domain mapping revealed a bimodal mechanism of action of Brd4: its N-terminal region containing the two bromo-domains and motifs “A” and “B” interact with nucleosomes, whereas its C-terminal region containing Extraterminal (ET) and SEED domains directly bind the C-terminal domain of MLV integrase. JQ-1, a small molecule that selectively inhibits interactions of BET proteins with their cognate modified histone sites impaired MLV but not HIV-1 integration in infected cells. Comparison of the distribution of BET protein binding sites analyzed using ChIP-seq data and MLV integration sites revealed significant positive correlations. Antagonism of BET proteins, via JQ-1 treatment or RNA interference, reduced the frequency of MLV integration at transcription start sites.

## Conclusions

Our findings demonstrate the importance of BET proteins for MLV integration efficiency and targeting, and provide a route to developing safer MLV-based vectors for human gene-therapy.
